# Editorial: Families and COVID-19: An Interactive Relationship

**DOI:** 10.3389/fsoc.2022.841518

**Published:** 2022-02-24

**Authors:** Linda Hantrais, Julia Brannen, Nicky Le Feuvre, Marie-Thérèse Letablier

**Affiliations:** ^1^International Inequalities Institute, London School of Economics and Political Science, London, United Kingdom; ^2^Social Research Institute, Institute of Education, University College London, London, United Kingdom; ^3^Faculty of Social and Political Science, University of Lausanne, Lausanne, Switzerland; ^4^Centre d'Economie de la Sorbonne, CNRS/Université Paris I, Paris, France

**Keywords:** family and household structures, impacts of COVID-19, policy responses, socio-economic inequalities, vulnerabilities

## Introduction

When the World Health Organisation (WHO, 2020) declared that Europe had become the epicentre of the COVID-19 pandemic in March 2020, the disease was recognised as a novel and virulent strain of coronavirus, presenting an unprecedented global threat to humanity, against which populations had no natural immunity. Comparisons with death rates in the Spanish flu pandemic in 1918 and both world wars showed how COVID-19 was affecting very different age groups (Spinney, 2017). Unlike earlier pandemics and global crises, older people with co-morbidities quickly became a focus for attention as the most vulnerable population category with the greatest likelihood of being hospitalised and dying from the disease.

Evidence was also sought from earlier pandemics about the most effective ways of controlling the spread of the virus. The relative success of East Asian countries in containing COVID-19 at its onset was widely attributed to their capacity to learn from previous experience of epidemics, their preparedness to deal with new threats to health, and public acceptance of the need to comply unquestioningly with stringent public health measures (Cairney and Wellstead, 2021). WHO and national governments were accused of being ill-prepared for a global pandemic, of reacting too slowly in closing their borders and then implementing stringent lockdowns that caused irreparable damage to the economy and to the livelihoods of families and communities (Boin et al., 2020; Capano et al., 2020).

Social science evidence collected in the early phase of the pandemic in Europe identified a wide range of socio-demographic, economic, political and environmental factors that were affecting vulnerability to the disease (Hantrais and Letablier, 2021). Countries, or areas within them, with densely populated, high urban concentrations and internationally connected populations, in conjunction with high old-age dependency ratios and high rates of underlying health conditions (obesity, diabetes), were more likely to record larger numbers of COVID-19 cases and deaths. Poorly funded and equipped public healthcare provision, and underdeveloped technological infrastructures, crowded living conditions in multigenerational households, risky lifestyles, and precarious working arrangements in low-paid public-facing jobs, particularly when carried out by ethnically diverse populations, compounded the risk of contracting and dying from the disease. The pandemic presented greater challenges for policymakers in regions where these underlying conditions were associated with entrenched socio-economic and political divisions, unstable or dysfunctional governments, skeptical electorates and hostile media.

Relatively little reliable information was available at this early stage in the pandemic about the differential impact of lockdown measures on the everyday lives and relationship of family members within and across societies. Nor was sufficient attention being devoted to variations in the impact of family composition and living arrangements on the transmission of the disease and its outcomes. Yet, social scientists were aware that existing socio-economic inequalities were being intensified because of the failure to focus on the effects at the micro-social level of lockdown measures, particularly school and workplace closures, restrictions on entertainment and public gatherings, and social distancing rules.

Evidence from small-scale sociological studies gradually raised awareness among policymakers of the indirect and potentially longer-term effects of the pandemic on the social, psychological and economic lives of households and families (Maor and Howlett, 2020). The accumulating international evidence base confirmed the importance for policy development of understanding the complex interactive relationship between socio-demographic and environmental health determinants on the transmission of the disease within households and of its etiology within different population groups (Hantrais and MacGregor, 2021).

The aim of this special issue of *Frontiers in Sociology* is to fill some of the gaps in knowledge about the interactive relationship between families and COVID-19, drawing on evidence collected in a wide range of cultural and disciplinary contexts. Contributors to the special issue examine similarities and differences in the experiences of families, especially during the first wave of the pandemic (see Table 1). They show how families and households served as a prism or social laboratory for studying the dynamics of everyday life and the ways that livelihoods were affected by policy measures as households became a critical site for government interventions.

**Table 1:** International and methodological perspectives on family life.

**Authors**	**Title**	**Country**	**Research design**	**Data collection**	**Data analysis**
Trotter	Ways of Being Together during the COVID-19 Pandemic: Support Bubbles and the Legal Construction of Relationships	New Zealand UK nations	Archival and contemporary material	None	Documentary
Shah et al.	Growing up under Covid-19: Young People's Agency in Family Dynamics	Lebanon Italy Singapore UK	Participatory approach (non-representative sample)	Digital and online interviews (small samples)	Qualitative analysis
Hu and Qian	COVID-19, Inter-household Contact and Mental Well-being among Older Adults in the US and the UK	US and UK	National and international longitudinal studies (representative samples)	Face-to-face and online national survey data	Secondary quantitative analysis
Palma and Araos	Household Coping Strategies during the COVID-19 Pandemic in Chile	Chile	National panel study (representative sample)	National survey data	Secondary quantitative analysis
Kutsar and Kurvet-Käosaar	The Impact of the COVID-19 Pandemic on Families: Young People's Experiences in Estonia	Estonia	Collaboration with schools and a national museum qualitative interviews (purposive sample)	Written diaries face-to-face/ online/ phone interviews	Qualitative analysis
Lambert et al.	Socio-economic Impacts of COVID-19 on Working Mothers in France	France	Wave of longitudinal online survey (national quota sample), ongoing qualitative study	Online survey and telephone/online interview data	Quantitative and qualitative analysis
Gouveia et al.	Household Diversity and the Impacts of COVID-19 on Families in Portugal	Portugal	Cross-sectional national survey (non-representative) and qualitative online study	Online survey and subset of online interviews	Quantitative and qualitative analysis
Wissö and Bäck-Wiklund	Fathering Practices in Sweden during the COVID-19: Experiences of Syrian Refugee Fathers	Sweden Syria	Longitudinal qualitative study	Face-to-face interview data	Qualitative analysis
Bühler et al.	Caring during COVID-19: Reconfigurations of Gender and Family Relations during the Pandemic in Switzerland	Switzerland	Qualitative study of selected groups drawn from an epidemiological project	Face-to-face interviews at home or in research institute	Qualitive analysis

Together the articles in the collection present an impressive body of quantitative and qualitative data about the interactive relationship between families and the pandemic in a large range of countries at the intersections between ethnicity, gender, age, health status, occupation, income and housing. They reveal the challenges that arise in assessing and brokering evidence about these interactions in different socio-economic and political settings. They also show how social science research evidence can be used to inform and shape policy, while mitigating impacts of the pandemic in the immediate and longer term, and improving outcomes for families in different policy settings. Although each of the studies is contextualised, few are explicitly comparative. But the evidence from this body of original theoretical and empirical analysis, in conjunction with the available literature that is cited, provides a robust basis for international comparisons of the interactive family–COVID-19 relationship.

## International and Disciplinary Perspectives

Most articles in the special issue focus on a single country, and a specific age or ethnic population group, or household types within it. But all the studies are contextualised in relation to research carried out in similar or contrasting socio-cultural, economic and political environments, thereby extending coverage of the topic within and beyond Europe, to OECD and African countries. The multi-national authors represent a variety of social science disciplines and subdisciplines, from family sociology, anthropology, demography to gender and socio-legal studies, social policy and social work.

As family sociologists in Chile, Palma and Araos examine the intergenerational coping strategies adopted by different types of households during the pandemic. Gouveia et al. describe the wider socio-economic context of Portuguese society that preceded the pandemic and the ways in which it exacerbated inequalities between households and families. The research by Hu and Qian, family sociologists based in the UK and Canada, is explicitly comparative in considering how the physical and virtual isolation imposed on older people in the UK and US during the pandemic affected their mental well-being.

Lambert et al. adopt a socio-demographic perspective in exploring the impact of COVID-19 in France on gender equality in households with children and how it reflected household living and working arrangements prior to the crisis as well as during lockdown. Bühler et al. conducted an anthropological analysis of the reconfiguration of gender and family relations in a Swiss canton to understand the interactive relationship between risk and protective practices. In Sweden, Wissö and Bäck-Wiklund drew on their backgrounds in social work to carry out a sociological analysis of the fathering practices of Syrian refugees.

Shah et al. engaged young people in four geographically and culturally different countries (Lebanon, Italy, Singapore, and the four UK nations) in participatory action research. Their investigation focused on young people‘s perceptions of growing up under COVID-19. Kutsar and Kurvet-Käosaar explored the experiences of children in Estonia within the broader context of the Baltic States from sociological and cultural perspectives.

By documenting the rules regarding support bubbles adopted in the four UK nations, following the example of New Zealand, Trotter's article brings a legal dimension to the analysis of the impact of government restrictions on family life.

## Methodological Approaches

The conditions during the pandemic hampered the process of carrying out empirical social science research. While the opportunity for primary research that involved face-to-face fieldwork became impossible, one of the major consequences of the pandemic on families was the growth of online practices, whether for children's education, parents' employment or communications between relations and friends. Such significant changes in everyday life had implications for how researchers carried out their trade. The rapidity with which people were forced to conduct their lives online offered an opportunity for social science to make a virtue out of necessity.

Most of the studies and analyses in the special issue were designed specifically to document the pandemic's effects on family lives (see Table 1). The original aim of the small-scale qualitative study carried out by Wissö and Bäck-Wiklund was different. Already in 2019, the authors had been examining the fathering practices of refugees as part of a comparative project in Sweden and the UK. The project's longitudinal design made it possible to approach the families again in 2020 to understand their everyday experiences during the pandemic. The study by Lambert et al. was also able to draw on pre-pandemic work, in their case on class, gender and generational inequalities in France, to capture changes in attitudes and practices. Authors of other dedicated studies, carried out as the pandemic progressed, questioned respondents and interviewees about their subjective perceptions of the changes that COVID-19 was bringing about in their lives.

Nearly all the articles in the special issue resorted to online data collection methods during the pandemic. Unlike the other authors, Trotter relied solely on documentary evidence assembled from a wide range of official and administrative sources in combination with political speeches, journalistic and other media reports, to track the development of support bubbles across time (from the onset of the pandemic to the submission date of the article) and space (from New Zealand to the four UK nations). Significantly, almost all her materials were available online.

The most ambitious example of original empirical online research, in terms of the sample size achieved, was the survey conducted by Gouveia et al. in Portugal. The research team publicised their survey through mainstream media and online platforms: websites, Twitter and Facebook accounts and email distribution lists. The final sample included 11,508 adults, but it was non-probabilistic, which limited statistical inference as well as an accurate representation of all segments of society. Biases were geographical, educational and social class related. For example, residents in the Greater Lisbon Area and large urban coastal areas were over-represented, as were respondents with a university degree, whereas those with routine manual and frontline service work were under-represented. To complement their quantitative data, Gouveia et al. selected and studied a subset of cases adopting a qualitative approach using online methods (Braun et al., 2020). By posing open-ended questions to a small sample drawn from a wider survey, the authors were able to exemplify the experiences and strategies adopted during the pandemic that had been identified in the survey data.

Digital methods and access contributed to similar biases in small-scale qualitative studies involving online sampling, which the authors sought to overcome by using a purposive selection of cases for in-depth analysis (Kutsar and Kurvet-Käosaar; Lambert et al.; Shah et al.). The article by Bühler et al. reports on a stand-alone qualitative study designed to complement the results from an epidemiological project on the transmission of, and immunity to, SARS-CoV-2, in a Swiss canton. The cases selected provide an in-depth description of how the respondents reconfigured their lives during the first lockdown. The mixed methods design employed by Lambert et al. enabled them to address temporal concerns. The national survey that they were analysing failed, however, to capture the experience of highly vulnerable groups, such as lone mothers who were particularly affected by the pandemic. Nor did it permit direct comparisons to be made between men and women within couples. The authors therefore used a panel of respondents who were being followed prior to, and during, the pandemic to complement the quantitative data from the national survey.

An obvious way for researchers to access large-scale background data relating to families' experiences of the pandemic was to draw on existing national datasets. Whether the analysis of findings from large-scale surveys constitutes “dataset re-use” or “secondary analysis” (Brannen et al., 2021) is a moot point given that some of these datasets were ongoing national panel studies that were adapted to include questions relating to COVID-19. The advantage of secondary analysis is that it involves bringing new research questions to existing data (Heaton, 2004). Building on pre-pandemic work on household coping strategies, Palma and Araos carried out secondary analysis of data from a new national panel survey collected by the Ministry of Social Development during the first wave of the pandemic in Chile to identify its social consequences for the living conditions of families. Hu and Qian undertook secondary analysis of data from two waves (post and pre-pandemic) of the US international longitudinal Health and Retirement Study and the UK national longitudinal study, Understanding Society, in their comparative analysis of the effects of virtual vs. face-to-face contact on the mental well-being of older adults.

Since researchers could not easily gain direct access to research participants, as in much primary social science research, they used intermediaries to recruit participants. In a study of young people's experiences of the pandemic, Kutsar and Kurvet-Käosaar exploited a public data collection campaign organised by the Estonian Literary Museum in 2020 to provide data for their archives. Schools were asked to give pupils an assignment in which they kept lockdown diaries or wrote memoirs during the first wave of the pandemic that would ultimately be placed in the museum's collection. A year later, during the second wave of the pandemic, the authors organised a fieldwork assignment for their university students designed to track changing reactions to the pandemic. The students were required to interview a small sample of children whom they knew using convenience sampling with purposive sampling elements. This method enabled the authors to gain insights into how attitudes and behaviours were evolving over a longer period of time.

Another way in which young people were encouraged to contribute to research about the impacts of the pandemic on their lives was through the use of participatory methods. Drawing on earlier work on family interdependencies, Shah et al. adopted a participatory ethnographic action approach. They invited young people in four countries (Italy, Lebanon, Singapore and the UK) to document their experiences of the pandemic. The co-researchers within and across the countries were organised into seven panels. Video calls and an online collaboration platform were supplemented by individual online interviews. Participation varied across the countries owing to differences in socio-economic conditions, in COVID-19 case numbers and in internet connectivity. High rates of attrition occurred, due in part to “zoom fatigue”, despite the engagement strategies employed by the core research team to build rapport with, and support, young people over time, including private messaging and one-to-one calls.

Although not explicitly discussed in the nine articles, the pandemic was found to have wrought major changes in the ways in which social science research is carried out: whether it be in data sources, collection and analysis, or the dissemination of findings. These changes beg the question about the longer-term impact of COVID-19 on social science and its methodologies.

## Families as Sites for Government Interventions

When Europe was recognised as the epicentre of the pandemic in March 2020, the prevailing climate of uncertainty was intensified by inconsistent scientific advice and intractable political dilemmas. Hotspots were identified for transmission of the virus, focusing on frontline workers, shielders for vulnerable members, rules for family gatherings and ceremonies, which were used to legitimise intrusions in family life and privacy. To be effective, social and physical distancing in work, play, education and everyday life, as well as limitations on mobility and access to family care, required compliance by family members and within families. Family members were also recipients of state support. Governments experimented with packages of measures based on limited and, at times, contradictory evidence about their effectiveness in preventing transmission of the disease and high excess death rates, amid growing concern about the collateral damage being caused to public health, and to social and economic life. The articles in the collection show how, in the competition for resources, politicians, policymakers and households faced moral and practical dilemmas. They document the intergenerational and gendered conflicts that arose, challenging both family and social solidarity.

National and localised lockdowns were introduced with different degrees of stringency. Sweden (Wissö and Bäck-Wiklund) and Switzerland (Bühler et al.) stand out as countries featured in the special issue with the most flexible or “softer” forms of lockdown, but without providing any tangible evidence that this flexibility eased pressures on family members. By leaving individuals to take personal responsibility for deciding whether to comply with the regulations, the contrary may even have been the case.

All the articles in the special issue address the issue of how the pandemic, mediated by government policies and, importantly, by access to digital technologies, affected living arrangements and intra-familial relationships, as the closure of public spaces and activities (for education, work and leisure) forced family members to spend more time at home together or apart. Trotter documents the many ways in which the law constructs and acts on perceptions of the experience of being together. By showing how support bubbles were defined and implemented in the four nations in the UK, she highlights regional differences in the extent to which the state sought to control relationships within families, resulting in confusion and difficulties for families whose members were dispersed across the country. Other articles identify disparities in the experiences of living through the pandemic associated with age and generation (Hu and Qian; Kutsar and Kurvet-Käosaar; Shah et al.), socio-economic status and gender (Bühler et al.; Lambert et al.), ethnicity and immigration (Bühler et al.; Wissö and Bäck-Wiklund), and living arrangements, particularly in countries with relatively large numbers of multigenerational households (Gouveia et al.; Palma and Araos).

Responses in surveys and interviews revealed how the closure of schools, offices, sporting and cultural venues impacted on the social and psychological well-being of young people. Several articles in the collection show how young people perceived the experience of lockdown as both negative and positive (Kutsar and Kurvet-Käosaar), and how they were able to play a role in managing the situation and asserting their right as active and autonomous participants in civic and social life (Shah et al.).

Not only did lockdown measures create opportunities to discover the benefits of closeness and a new understanding between the generations, but they also uncovered latent tensions and conflicts within households (Gouveia et al.; Kutsar and Kurvet-Käosaar). Gender differences were starkly exposed, as traditional gender roles (caring, family–work reconciliation) were simultaneously reinforced and challenged (Bühler et al.; Lambert et al.). Analysis of the differential impact of the pandemic and associated lockdowns on gender relationships reveals different coping strategies and role adaptations of women and men during the pandemic (Lambert et al.), as well as their capacity to develop effective income-generating and expenditure-minimising coping strategies (Palma and Araos). The findings confirm that women bore the main brunt of lockdowns, even if men temporarily assumed a shielding role to protect their families from viral exposure (Bühler et al.).

Although national media, and several of the articles in the collection, focused on the negative impact of the restrictions on the mental health of young people, the article by Hu and Qian compared the effects of policy measures on the mental health of older people in the UK and US. The authors found that virtual contact, including by telephone, was not a qualitatively equivalent alternative to face-to-face contact. This finding also applied to young children in Estonia (Kutsar and Kurvet-Käosaar). In Chile, by contrast, older people were found to suffer less financial hardship than other age groups due to their relatively secure, albeit very low, incomes from pensions in a context where state welfare support for workers and their families was limited (Palma and Araos).

Studies that adopted a longitudinal perspective (Lambert et al.), and/or questioned respondents about the changes they had been forced to make to their daily lives (Bühler et al.; Gouveia et al.; Kutsar and Kurvet-Käosaar; Palma and Araos), revealed growing socio-economic inequalities in the opportunities and threats presented by the pandemic (Wissö and Bäck-Wiklund). Restrictions on access to education and workplaces was a central concern, particularly with the sudden shift to online communications (teleworking and distance learning). Studies that tracked changes over time showed how public attitudes and behaviours evolved as governments progressively — often too rapidly — relaxed restrictions to relieve economic hardship, by lifting travel bans and reopening hospitality, entertainment and tourism sectors, schools and offices. Respondents reported that they were suffering from pandemic fatigue and were becoming less willing to comply with stringent lockdown measures (Gouveia et al.; Kutsar and Kurvet-Käosaar; Lambert et al.).

In addition to tracking the impact of government measures on the experiences of family members, the contributors show how, to varying degrees, governments listened to the accumulating social science evidence in formulating policy responses to the many challenges they were facing (Kutsar and Kurvet-Käosaar). Findings from the studies emphasise the importance of providing multilevel welfare interventions to ensure that they cater for differentiated social needs and vulnerabilities (Gouveia et al.; Palma and Araos; Wissö and Bäck-Wiklund); that they mitigate the unexpected and less visible and unequal impacts of the pandemic on everyday lives (Bühler et al.; Hu and Qian; Lambert et al.; Shah et al.); and that they improve outcomes for families in different policy contexts within and between countries in the immediate and longer term.

## Conclusions

Although the contributions to the special issue have a number of limitations concerning both spatial and temporal coverage, in combination, they provide a robust international evidence base for methodological and theoretical analyses of the complex interactive relationship between families (and households) and COVID-19 as mediated through public policy.

Through the lens of the pandemic, the authors contribute empirically to the anthropological, sociological and social policy literature on family practices concerning parenting roles, intergenerational responsibilities for care, the gendered division of tasks, life-course and linked lives trajectories and subjective perceptions of vulnerability, risk and resilience. Their cumulative findings documenting direct experiences of a severe and unexpected social phenomenon clearly demonstrate the importance of the ways in which pre-existing social vulnerabilities and inequalities were exacerbated by the pandemic as well as the value for policy development of recognising the multidimensionality of its material and subjective impacts in socially differentiated contexts.

## Author Contributions

All authors listed have made a substantial, direct, and intellectual contribution to the work and approved it for publication.

## Conflict of Interest

The authors declare that the research was conducted in the absence of any commercial or financial relationships that could be construed as a potential conflict of interest.

## Publisher's Note

All claims expressed in this article are solely those of the authors and do not necessarily represent those of their affiliated organizations, or those of the publisher, the editors and the reviewers. Any product that may be evaluated in this article, or claim that may be made by its manufacturer, is not guaranteed or endorsed by the publisher.
